# Anticoagulation Therapy in a Patient With Heterozygous Factor V Leiden and Coexisting Homozygous Prothrombin Gene Mutations

**DOI:** 10.7759/cureus.11949

**Published:** 2020-12-07

**Authors:** Ryder L Costa, Molly Triggs, Shelbie E Cole, Joshua Lacey, Samarth Reddy

**Affiliations:** 1 Research, Alabama College of Osteopathic Medicine, Dothan, USA; 2 Hematology and Oncology, Hematology Oncology Associates of Boca Raton, Boca Raton, USA

**Keywords:** pulmonary embolism (pe), heterozygous factor v leiden mutation, prothrombin

## Abstract

Coexistent heterozygous factor V Leiden and homozygous prothrombin G20210A gene mutations is a rare and potentially life-threatening occurrence. This inherited thrombophilia often presents as non-specific venous thromboemboli and can mimic a variety of emergent medical conditions. The pathophysiology of the disease has been well documented; however, long-term treatment efficacy remains poorly understood. We report the case of a 25-year-old male presenting with acute chest pain. A comprehensive workup revealed bilateral pulmonary emboli arising in part from concomitant heterozygous factor V Leiden and homozygous prothrombin G20210A gene mutations. Immediate and continuous treatment with anticoagulants enoxaparin and apixaban significantly reduced the patient’s symptoms and D-dimer within one week. This case provides insight into an effective treatment regimen for this rare and inherited thrombophilia.

## Introduction

The prothrombin G20210A gene mutation is a common coagulopathy that impacts 1-4% of the U.S. and European population and is inherited in an autosomal dominant fashion [1]. A point mutation from guanine to adenine causes increased prothrombin levels in homozygotes by 70% [2]. This excess prothrombin increases the blood propensity to clot. Factor V Leiden (FVL) is an autosomal-dominant inherited thrombophilia. The heterozygous genotype of FVL mutation impacts 3-8% of the U.S. and European population [3]. In contrast to prothrombin G20210A, FVL leads to an increased resistance of factor V to inhibition by protein C, thereby creating a hypercoagulable state [4]. Both of these diseases typically present as venous thromboembolism in varying locations [5]. Treatment of these distinctly different coagulopathies in a single patient can be challenging given the paucity of literature. Here, we report the case of a 25-year-old male with coexistent homozygous prothrombin G20210A mutation and heterozygous FVL mutations who was effectively treated with an anticoagulant regimen consisting of enoxaparin and apixaban.

## Case presentation

A 25-year-old Caucasian male presented to the emergency department with a one-day history of left-sided chest and back pain that radiated to the left scapula. The pain existed at rest and increased with inspiration. He had no present complaints of leg pain. The patient reported a three-month history of cough and shortness of breath. He had no significant medical conditions. The patient had no prior history of coagulopathic events, such as deep vein thrombosis (DVT). His family history was significant for transient ischemic attacks, but there was no history of early cardiovascular events.

Physical examination revealed a well-nourished, college-aged male in obvious distress. Vital signs showed tachycardia (120 bpm), a respiratory rate of 22 breaths per minute, and sufficient oxygen saturation on room air (SpO_2_ of 99%). The patient’s lungs were clear to auscultation. Breath sounds were equal, and symmetrical chest wall expansion was observed. Normal S1 and S2 heart sounds were appreciated with elevated rate and regular rhythm. Abdominal examination revealed no acute findings. There were no indications of peripheral edema, and Homan’s sign was negative.

**Figure 1:**
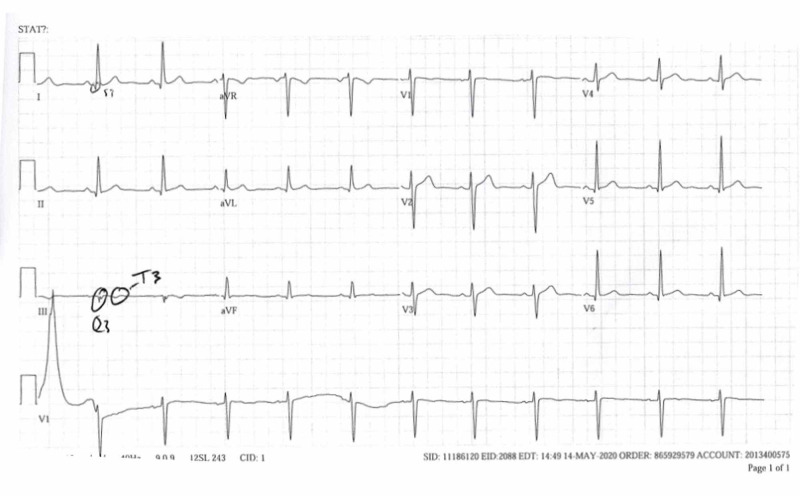
ECG with S1Q3T3 pattern consistent with pulmonary embolism.

**Figure 2:**
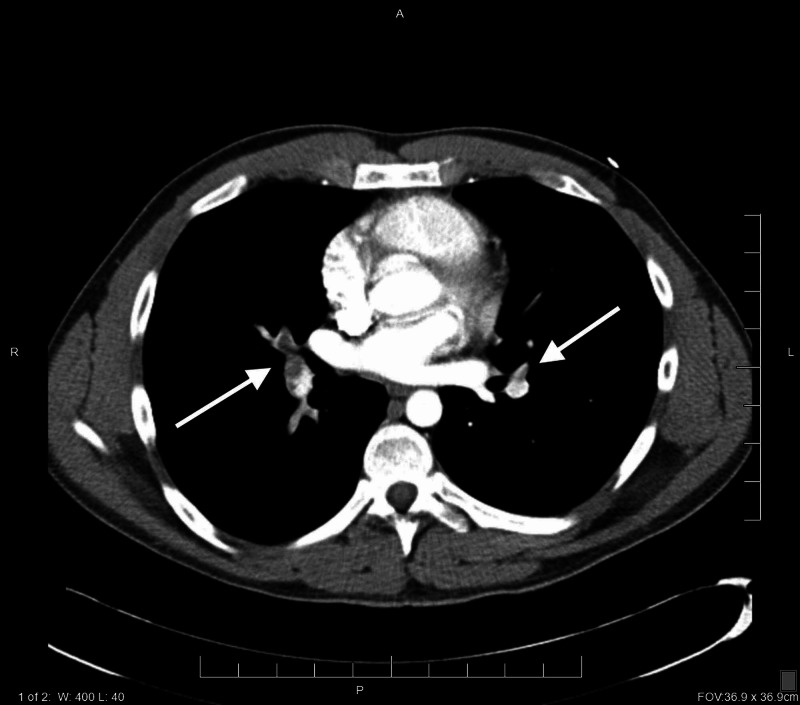
CT angiogram demonstrating filling defects in branches of pulmonary arteries bilaterally in keeping with pulmonary emboli.

Electrocardiogram (ECG) was performed upon admittance, which showed sinus tachycardia and nonspecific T and ST wave abnormalities (Figure 1). Initial serology showed hypokalemia (3.5 mmol/L; reference range: 3.6-5.2 mmol/L), elevated D-dimer (1.79 mg/L; reference range: 0.00-0.49 mg/L), and troponin within normal range (<0.015 ng/mL). A computed tomography angiogram (CTA) of the chest was performed, which indicated pulmonary emboli (PE) in both lower lobes and the right middle lobe (Figure 2). Ground-glass opacities were also noted in the left lower lobe. There were no indications of right-sided heart strain, aortic dissection, or aneurysmal dilation. A CT of the abdomen and pelvis were negative for malignancy. Ultrasound imaging of bilateral lower extremities was negative for evidence of DVT, and the patient was negative for COVID-19 after a nasal swab was performed. Coagulation studies were negative for protein C, protein S, and antithrombin III deficiency, and lupus anticoagulant. Polymerase chain reaction (PCR) was positive for heterozygous FVL and homozygous for prothrombin G20210A mutation.

Given the suspected diagnosis of PE, the patient was administered two doses of enoxaparin 90 mg subcutaneously and was admitted overnight for observation. After 48 hours, the patient was discharged from the hospital and prescribed apixaban 10 mg daily. After 10 days, the patient’s D-dimer had decreased from 1.79 mg/L to 0.53 mg/L. The patient was prescribed regular apixaban (5 mg twice daily) with yearly evaluation of D-dimer levels due to the unprovoked PE. After one week, the patient was able to resume his activities of daily living and has not experienced a recurrence of thromboembolic events.

## Discussion

Prothrombin G20210A and FVL mutations are considered common thrombophilias independently. However, the presence of concomitant heterozygous FVL and homozygous prothrombin G20210A mutations is exceedingly rare, only occurring in roughly one out of 200,000 persons worldwide [6]. Patients with this rare condition are at a 17-fold higher risk of developing venous thromboembolism (VTE) than patients without this genotype [7]. Nevertheless, the lack of research into effective treatment for this genotype presents treatment challenges and a gap in the literature. This case describes the efficacy of immediate and long-term anticoagulant treatment in a patient with symptomatic homozygous prothrombin G20210A and heterozygous FVL mutations.

A genetic thrombophilia should be considered when a patient under 50 years of age develops a VTE [7]. If a VTE is suspected, a D-dimer test and CT can be useful in the diagnosis. After confirming the presence of a VTE, genetic testing by PCR is recommended to screen for inherited thrombophilias. In this case, the patient’s D-dimer was elevated at 1.79 mg/L, and a chest CTA showed bilateral and right middle lobe PE. Given the patient’s young age and lack of morbidities, we elected to pursue genetic testing. Different inherited thrombophilias and different genotypes of those thrombophilias present particular risks for recurrent VTE [7]. Therefore, it is critical to determine what mutation is causing the coagulopathy.

The rarity of a homozygous prothrombin combined with a heterozygous FVL mutation poses new challenges in treatment options; however, research suggests that long-term use of apixaban can prevent the recurrence of VTE [6,8]. A case series showed that out of 100 patients with compound FVL mutation and prothrombin G20210A mutation, 68 experienced a VTE, and 25 of those patients experienced at least one recurrent VTE [6]. However, 72% of the patients with recurrent VTE were not receiving anticoagulants at the time of recurrence [6]. These data suggest that patients with thrombophilias should be on long-term anticoagulant therapy, such as apixaban. Moreover, another clinical trial illustrated that indefinite anticoagulation therapy reduces the risk of recurrent VTE in all subgroups of patients with idiopathic VTE, thereby suggesting this therapy should be the standard of care in the absence of contraindications [8].

In this case, we elected to prescribe enoxaparin acutely and apixaban indefinitely. Enoxaparin works to reduce further clot formation by stimulating antithrombin activity [9]. Apixaban directly inhibits factor Xa and inhibits prothrombinase activity, leading to decreased clot formation [10]. After one month of therapy, the patient’s D-dimer level dropped from 1.79 mg/L to <0.20 mg/L. The patient’s symptoms subsided in accordance with the reduction in D-dimer, and he was able to resume his activities of daily living without restrictions. The patient will receive periodic evaluation of D-dimer levels to monitor and prevent the recurrence of VTE. This treatment option should be considered in the presence of unprovoked VTE.

## Conclusions

Treatment strategies for patients with concomitant heterozygous FVL and homozygous prothrombin G20210A mutations are not well documented in the literature. Here, we documented the case of a 25-year-old male with this rare combination of coagulopathies who presented with bilateral PE. Immediate and indefinite anticoagulant therapy resulted in a reduction in symptoms and D-dimer levels and a return to everyday living. This case report supports the use of enoxaparin and apixaban therapy as an effective treatment solution in patients with similar characteristics.

## References

[1] Shemesh A, Hoffman R, Nadir Y, Keren-Politansky A, Monreal M, Brenner B, Tzoran I (2017). Clinical significance of prothrombin G20210A mutation in homozygous patients. Am J Hematol.

[2] Segal JB, Brotman DJ, Necochea AJ (2009). Predictive value of factor V Leiden and prothrombin G20210A in adults with venous thromboembolism and in family members of those with a mutation: a systematic review. JAMA.

[3] Dautaj A, Krasi G, Bushati V (2019). Hereditary thrombophilia. Acta Biomed.

[4] Kumar V (2015). Hemodynamic disorders, thromboembolic disease, and shock. Robbins and Cotran Pathologic Basis of Disease, Ninth Edition.

[5] Kovac M, Mitic G, Mikovic Z (2010). Type and location of venous thromboembolism in carriers of factor V Leiden or prothrombin G20210A mutation versus patients with no mutation. Clin Appl Thromb Hemost.

[6] Lim MY, Deal AM, Kim S (2016). Thrombophilic risk of individuals with rare compound factor V Leiden and prothrombin G20210A polymorphisms: an international case series of 100 individuals. Eur J Haematol.

[7] Hotoleanu C (2017). Genetic risk factors in venous thromboembolism. Adv Exp Med Biol.

[8] Goldhaber SZ (2004). Prevention of recurrent idiopathic venous thromboembolism. Circulation.

[9] Siddiqui MA, Wagstaff AJ (2005). Enoxaparin: a review of its use as thromboprophylaxis in acutely ill, nonsurgical patients. Drugs.

[10] Byon W, Garonzik S, Boyd RA, Frost CE (2019). Apixaban: a clinical pharmacokinetic and pharmacodynamic review. Clin Pharmacokinet.

